# The Correlation of Age and Postoperative Visual Acuity for Age-Related Cataract

**DOI:** 10.1155/2016/7147543

**Published:** 2016-01-05

**Authors:** Xiaochun Li, Xiaoguang Cao, Xianru Hou, Yongzhen Bao

**Affiliations:** ^1^Department of Ophthalmology, Peking University People's Hospital and Key Laboratory of Vision Loss and Restoration, Ministry of Education and Beijing Key Laboratory for the Diagnosis and Treatment of Retinal and Choroid Diseases, Beijing 100044, China; ^2^Department of Ophthalmology, Peking University International Hospital, Beijing 102206, China; ^3^People Eye Center, Peking University People's Hospital, No. 11 Xizhimen South Street, Xicheng District, Beijing 100044, China

## Abstract

*Purpose*. Clinically, what is the best time for age-related cataract (ARC) patients to receive surgeries and get the most benefits is important. We explored the relationship between age and presenting postoperative visual acuity (POVA) in patients from rural China.* Methods*. Three Lifeline Express Hospital Eye-Train missions of Peking University People's Hospital were chosen. At the first day after surgery, 3452 ARC eyes with the presenting POVA ≥ 6/60 were enrolled. The relationship between age and POVA was analyzed statistically.* Results*. In these three missions, there were more female patients than males; the ratio of females to males was 1.71. The average age of females was older than males. Overall, the percentages of patients with good visual outcomes (≥6/18) were significantly decreased with aging. Different regions had variations, but the trends were the same. There was weak linear correlation between age and POVA. The correlations of females were stronger than males in Yuncheng and Sanmenxia and weaker than males in Zhoukou.* Conclusion*. The good visual outcomes of presenting POVA were significantly decreased with aging and there were weak linear correlations between age and POVA in rural China. The linear correlation might be influenced by the difference of gender and region.

## 1. Introduction

According to China's Ministry of Health, China has approximately 4 million people that suffered from cataract, with 500,000 new cases being diagnosed each year. As a developing country, especially in rural China, the distribution of health care resources is uneven due to poverty. Lots of patients have limited access to health care and have great difficulty in obtaining proper treatment. In 1997, with the support from the Ministry of Health, the Ministry of Railways, and the Hong Kong and Macao Affairs Office in Mainland China, Lifeline Express Hospital Eye-Train (the Eye-Train, LEHET) first embarked on its mission of light into China's countryside, as a gift to the people of Mainland China, proving charge-free cataract surgeries. With new trains being commissioned in 1999, 2002, and 2009, there are now four Eye-Trains in continuous operation, which together cure more than 12,000 cataract patients each year. From 1997 to 2010, over 110,000 cataract patients received free surgery treatment. This not only has enormously changed each patient's own life, but also has brought hope and joy to their whole family [1].

Independent of cost or free charge, providing high quality cataract surgical services is critical for patients with cataract who are willing to have their sight restored. The visual acuity improvement after surgery is the key standard to evaluate how successful the surgery is; after cataract surgery, the visual acuity is measured predominantly. Because of poor health awareness and long way to hospitals (hundreds of miles away), few postoperative patients who had charge-free cataract surgery on LEHET could come back for follow-up eye examination. The visual acuity and other eye examinations performed at first day after operation were the only medical records on LEHET. So the first-day presenting postoperative visual acuity (POVA) was the only way to evaluate the outcome of cataract surgeries and the satisfaction of patients on LEHET.

Many factors could have influence on POVA. In the aging society in China, more and more people pay attention to the quality of life and try to extend their work life. The question “what age is the best opportunity to have the cataract surgery” is always being asked by patients, even doctors. The relationship between age and POVA in the age-related cataract (ARC) patients could be helpful to answer the question. Awareness of the relationship should be useful to clinicians when consulting elderly patients preoperatively to maintain realistic expectations of their surgery.

In this study, we explore the correlation of age and POVA in ARC patients in three missions, who received cataract surgeries on LEHET, in rural China.

## 2. Methods

### 2.1. Recruitment of Patients, Preoperative Assessment, and Inclusion and Exclusion Criteria

Our hospital, Peking University People's Hospital (PUPH), had four missions of LEHET, Zhoukou in Henan province and Songyuan in Jilin province in 2011, Yuncheng in Shanxi province, and Sanmenxia in Henan province in 2012. The sites were selected by the office of LEHET and kept unknown to our hospital before the mission starting. Songyuan in Jilin province was excluded from this study due to the incomplete data. Yuncheng (N 35.03; E111.01; altitude: 369.53 m) in Shanxi province and Zhoukou (N 33.62; E114.66; altitude: 50.50 m) in Henan province have thousands of years of history. Most residents in these two cities are rural people and live there since being born. Sanmenxia (N 34.77; E111.20; altitude: 376.08 m) in Henan province was built in the 1950s and also a rural city. Residents in this new city partly emigrated from the other areas of China, such as Northeast China and West China. In 2011, the average annual income per rural people was ¥5601.40 ($889.11) in Shanxi province and ¥6604.03 ($1048.26) in Henan province, much lower than Beijing ¥14735.68 ($2339.00) [2]. Based on the Sixth National Census of China 2010 (http://www.stats.gov.cn/) [3] and the 2010 annual survey data of China Disabled Persons' Federation (http://www.cdpf.org.cn/) [4], cataract surgical rate (CSR) was calculated as in Table 1.

Any patients who wanted to have the charge-free cataract surgeries on LEHET needed to register in the base hospital (a local hospital selected by the office of LEHET) first. After the systemic and ocular medical examinations and signed informed consent at the base hospital, the patients were sent to LEHET. Preoperatively on LEHET, all patients underwent a complete ophthalmological examination, that is, measurement of presenting visual acuity (VA) by means of Snellen charts (performed by the nurses from the base hospital), intraocular pressure (IOP) evaluation by noncontact tonometer (Canon TX-10/TX-F, Tokyo, Japan), slit lamp examination (Topcon SL-1E, Tokyo, Japan), and fundus examination (90 Dioptre, Volk Optical, Mentor, OH) with dilated pupil. Corneal curvature was measured by auto Keratometer (Nikon Speedy-K, Tokyo, Japan). Axial length (AL) measurement and B-scan were performed by ultrasonic system (ODM-2100, MEDA, Tianjin, China). Corneal endothelial counting (CEC) was performed by Specular Microscope (Topcon SP-3000P, Tokyo, Japan). The SRK/T formula for normal or long axial length (AL more than 25.00 mm) and Hoffer Q formula for short axial length (AL less than 22.00 mm) were used to calculate the power of intraocular lens (IOL) and the estimated postoperative refractive errors were less than ±0.25 D except patients with high myopia. Specular Microscope SP-3000P was equipped on LEHET since the second quarter of 2012, so the patients of Zhoukou and part of Yuncheng had no CEC measurement.

Inclusion criteria for this study are ARC patient with first-day POVA equal to or more than 6/60 without the macular diseases (except drusen) in the pre- and postoperative fundus examination.

Exclusion criteria for this study are age less than 50 years, axial length more than 25.0 mm, secondary cataract, previous intraocular surgery and corneal refractive surgery, ocular trauma, corneal degeneration and dystrophy (except arcus senilis), optic nerve disease, macular or retinal disease, severe systemic disease (such as uncontrolled hypertension or diabetes mellitus), and operative complications (e.g., posterior capsular rupture, vitreous loss, lost nucleus, zonule dehiscence, IOLs not in bag, sulcus fixation, and wound leak).

The study was in accordance with the tenets of the Declaration of Helsinki and has been approved by the Institutional Review Board of our hospital, PUPH. Written informed consent was obtained from all patients.

### 2.2. Description of the Procedure

The surgeons all came from our hospital, PUPH, and had titles of attending doctors or senior with two or three surgeons for each mission. Combined phacoemulsification and IOL implant (Phaco + IOL) were performed on 94.58% and combined sutureless, small-incision extracapsular cataract extraction and IOL implant (SIECCE + IOL) was performed on 5.42% patients by using Infinity Vision System (Alcon Laboratories). The 5.5 mm scleral tunnel incision was made for all patients when unfoldable PMMA IOL with 5.5 mm diameter of optic part and 12.5 mm length was implanted (PC55125, EYEGOOD (Zhuhai) Medicals Co., Ltd., China).

### 2.3. First-Day Presenting Postoperative Visual Acuity (POVA) Measuring

At Zhoukou in Henan province, POVA was measured on LEHET. At Yuncheng in Shanxi province and Sanmenxia in Henan province, POVA was measured in the base hospitals. The presenting POVA with Snellen charts was measured within half hour after removing the eye pad from operated eye. The measuring was independent of the surgeons and performed by the nurses that came from the base hospitals.

### 2.4. Statistical Analysis

Student's *t*-test was used to compare age and chi-square test was used to compare the female ratio and visual outcome between the groups. The linear regression was used to evaluate the correlation of age and POVA. A *p* value of less than 0.05 was considered to be statistically significant. Statistical analysis was performed using Statistical Product and Service Solutions software (SPSS version 20.0; Armonk, New York, USA).

## 3. Results

The demographic characteristics of the three missions are shown in Table 2. 3452 cataract patients (3452 eyes) were enrolled in this study, including 1274 males and 2178 females (male: female = 1: 1.71), and 1801 right and 1651 left eyes. Average ages of these cataract patients were 70.04 ± 7.10 years in males and 70.24 ± 6.95 years in females (*p* = 0.034). In different areas the average ages were slightly different. In Zhoukou, the average age of males was 69.04 ± 7.29 and 70.27 ± 6.94 in females (*p* = 0.012). In Yuncheng, it was 69.36 ± 7.33 in males and 70.70 ± 6.61 in females (*p* = 0.002). In Sanmenxia, it is 70.43 ± 7.31 in males and 69.81 ± 7.24 in females (*p* = 0.131). The average age of female is older than male's except in Sanmenxia. There were no statistically significant differences between missions preoperatively in age, gender, and operated eye between these three missions. POVA of Sanmenxia was better than Zhoukou and Yuncheng (*p* = 0.000 and *p* = 0.000, Student's *t*-test, resp.).

Table 3 showed the POVA according to age and gender. In all three missions, the percentages of good visual outcomes (equal to or better than 6/18) were decreased with aging (*p* = 0.000~0.003). In the male patients, there was no significant difference between average ages of the three missions (*p* = 0.092–0.212), the percentages of good visual outcomes decreased with aging (*p* = 0.005) in all males. In the female patients, the good visual outcomes decreased with aging not only for all females (*p* = 0.000), but also for the females in Zhoukou (*p* = 0.023) and Sanmenxia (*p* = 0.001), except those in Yuncheng (*p* = 0.085).

The calculation of linear regression is shown in Table 4. As *F* values are 61.522–70.312 and *p* values of ANOVA all are less than 0.001, there are linear correlations between age and POVA for these three missions. But as *R* values are less than 0.4, the linear correlations are weak.

As mentioned above, the average age of females was older than males'. The correlation of age and POVA was divided by gender and shown as in Tables 5 and 6. The same trends were indicated in the males and females that there were also weak linear correlations between age and POVA for each gender. The linear correlation of females is stronger than that of males in Yuncheng and Sanmenxia and on the contrary in Zhoukou.

## 4. Discussions

In the three missions, different surgeons performed the operations. The surgery outcomes between different missions might be influenced by the different experiences of the different surgeons. Furthermore, different regions and populations might have some unknown factors to influence the surgery outcomes. So we analyzed the data separately in different missions. We also put all the data from the three missions together to get the overall results.

Although, it has been found that there is a gender inequality in the uptake of cataract services where women are disadvantaged [5]. In our study, the patients were registered in the base hospitals with their wishes. Whether or not the patients were suitable for surgery was decided by the surgeons, dependent on the condition of eye, not the gender, although it has been found that there is gender unequal opportunity in the receiving of cataract surgeries because women are disadvantaged in rural areas of China [5]. The surgery was totally free of charge to all cases. The enrollment of patients in our study has nothing to do with genders or cost. The reasons why there were more female patients than male patients in all the three missions were unknown.

The good presenting POVA (equal to or better than 6/18) was 87.14% in all the patients and 83.61%, 86.69%, and 89.85% in patients from Zhoukou, Yuncheng, and Sanmenxia, respectively. In patients from Sanmenxia, the good presenting POVA was significantly higher than that of Zhoukou (*p* = 0.000) and Yuncheng (*p* = 0.015). The percentages of patients with the good presenting POVA (equal to or better than 6/18) in our three missions were much higher than that in patients with pseudophakics in other missions reported previously. It is even equal to or better than those POVA (equal to or better than 6/60) from some previous studies, such as in Jiangxi, China (64.56%–81.11%) [6], Malawi (60.42%) [7], Kenya (78.85% and 38.46%) [8, 9], Cameroon (54.55%) [10], Guatemala (66.35%) [11], Turkmenistan (71.64%) [12], Nepal (71.61%) [13], Pakistan (76.67%) [14], India (29.23%–81.00%) [15–17], and the Philippines (81.76%) [18, 19]. Compared with that presenting POVA reported for who was pseudophakics in some previous studies, these (89.87% and 85.54% for male and female, resp.) in our study were higher than that of the Philippines (80.77% and 82.61%, resp.) in each gender [18], lower than in Beijing (93.10% and 90.28%, resp.) [20]. The difference in presenting POVA compared with those previous studies could be due to the difference in proportion of cataract extraction methods and incision length. As had quite high components of aphakia, the cataract surgery data of those previous studies might be based on intracapsular cataract extraction (ICCE) and ECCE, which would lead to longer incisions and higher corneal astigmatism. Compared with phacoemulsification with a 3 mm or smaller wound, a high prevalence of significant postoperative astigmatism could be induced by ICCE (equal to or more than 2 D of astigmatism in 14%–58%) [21], ECCE (mean 1.6–3.5 D depending on technique and time after surgery) [22–24], and sutureless, manual cataract extraction (2.07 D at approximately 1 year) [25]. As 5.5 mm scleral tunnel incision was used, the astigmatism in our studies might be similar to some sutureless, manual cataract extraction or phaco surgery, around 1 D [26–28]. As evident, presenting POVA equal to or better than 6/18 was reported in Karki et al.'s study, 26.30% and 76.27% on first postoperative day for ECCE and small-incision cataract surgery (SICS), then 81.37% and 94.86% at 6-week postoperation for ECCE and SICS, respectively [29]. There is nothing to surprise that the visual outcome in our study was better than most of previous studies as Phaco + IOL was the predominant technique in cataract surgeries on LEHET now.

Increasing age is the most important risk factor for the development of cataract [30, 31]; the influence of increasing age on postoperative visual acuity for cataract had been previously documented as risk factor [32–35]. And previous studies indicated the age-related changes of postoperative visual acuity were statistically significant [36]. Controversially, one study reported the best-corrected visual acuity (BCVA) in 6 weeks after SICS positively associated with older age [37]. However, the studies have looked only at very broad age groups, such as 60–69-year-old and 70–79-year-old ones. Our study reported there was a linear correlation between age and POVA; even the correlation was weak. There are some possible reasons to explain the correlation. First, with aging, the cataract patients have more chance to have coexisting eye disease, such as age-related disorders, for example, macular degeneration. When the maculae look “normal” in fundus examination with dilated pupil, the mild macular disorders might already have influence on the function of fovea. Moreover, these disorders might be covered by the clouded lens. The second ones are the aging changes of nervous system. The accumulation of an autofluorescent pigment called lipofuscin in neurons is an invariable hallmark of brain aging. And lipofuscin in the macular retinal pigment epithelium (RPE) and choroid also increased with aging [38]. Moreover, the thickness of retinal nerve fiber layer was decreased with increased age [39]. These aging changes in the eye and brain might have influences on the visual acuity even with the clear media in the operated eyes. The third one is the severity of nuclear cataract. Usually, the density of nuclear cataract is increased and the corneal endothelium density is decreased with aging. As phacoemulsification was the main method of cataract extraction in our study, the corneal edema and incision burn might become severe with high power output of phacoemulsification. And that would have influence on POVA in our studies.

Overall, the linear correlations between age and POVA are similar in the males and females, in different missions; the correlations in the females were stronger than that in the males in Yuncheng and Sanmenxia and weaker in Zhoukou. The difference of correlation strength might indicate the correlation between age and POVA was influenced not only by the genders, but also by the different regions, as the distance between Yuncheng and Sanmenxia is near, low to 25 miles, although separated by the Yellow River.

## Figures and Tables

**Table 1:** CSR of China 2010.

Province	Population	Cataract surgeries	Charge-free cataract surgeries for low-income people	CSR	Charge-free CSR	Ratio of charge-free cataract surgeries for low-income people
Beijing	19612368	11961	1402	610	71	11.72%
Shanxi	35712101	15902	4929	445	138	31.00%
Henan	94029939	36935	9915	393	105	26.84%

CSR: cataract surgical rate.

**Table 2:** Demographic characteristics of the three missions.

	Zhoukou (*n* = 891)	Yuncheng (*n* = 1202)	Sanmenxia (*n* = 1359)	*p*
Age (years)	69.82 ± 7.09	70.21 ± 6.91	70.04 ± 7.27	0.467

POVA (LogMAR)	0.32 ± 0.25	0.31 ± 0.23	0.26 ± 0.23	0.000

Sex male/female	326/565	442/760	506/853	0.946

Operated eye right/left	494/397	625/577	682/677	0.050

**Table 3:** First-day postoperative visual acuity (POVA).

Regions	Gender	Age range (years)	Equal to or better than 6/18 *n* (%)	Worse than 6/18 *n* (%)
Zhoukou, Henan province	Male	50–59	33 (91.67%)	3 (8.33%)
		60–69	106 (90.60%)	11 (9.40%)
		70-	146 (84.39%)	27 (15.61%)
	Female^*∗*^	50–59	48 (90.57%)	5 (9.43%)
		60–69	147 (85.47%)	25 (14.53%)
		70-	265 (77.94%)	75 (22.06%)
	Sum^*∗*^		745 (83.61%)	146 (16.39%)

Yuncheng, Shanxi province	Male	50–59	50 (96.15%)	2 (3.85%)
		60–69	133 (92.36%)	11 (7.64%)
		70-	214 (86.99%)	32 (13.01%)
	Female	50–59	42 (93.33%)	3 (6.67%)
		60–69	216 (87.10%)	32 (12.90%)
		70-	387 (82.87%)	80 (17.13%)
	Sum^*∗*^		1042 (86.69%)	160 (13.31%)

Sanmenxia, Henan province	Male	50–59	45 (100.00%)	0 (0.00%)
		60–69	139 (91.45%)	13 (8.55%)
		70-	279 (90.29%)	30 (9.71%)
	Female^*∗*^	50–59	82 (96.47%)	3 (3.53%)
		60–69	258 (92.14%)	22 (7.86%)
		70-	418 (85.66%)	70 (14.34%)
	Sum^*∗*^		1221 (89.85%)	138 (10.15%)

Total of three regions	Male^*∗*^	50–59	128 (96.24%)	5 (3.76%)
		60–69	378 (91.53%)	35 (8.47%)
		70-	639 (87.77%)	89 (12.23%)
	Female^*∗*^	50–59	172 (93.99%)	11 (6.01%)
		60–69	621 (88.71%)	79 (11.29%)
		70-	1070 (82.63%)	225 (17.37%)
	Sum^*∗*^		3008 (87.14%)	444 (12.86%)

^*∗*^ *p* < 0.05 between three age ranges with chi-square test.

**Table 4:** Correlation of age and POVA.

		Zhoukou *n* = 891	Yuncheng *n* = 1202	Sanmenxia *n* = 1359	Total *n* = 3452
Correlations	Pearson	0.271	0.221	0.221	0.232
	*p*	0.000	0.000	0.000	0.000

*R*		0.271	0.221	0.221	0.232
*R* ^2^		0.073	0.049	0.049	0.054

ANOVA	*F*	70.312	61.522	69.588	196.721
	*p*	0.000	0.000	0.000	0.000

Coefficients	Constant	−0.335	−0.193	−0.218	−0.238
	*B*	0.009	0.007	0.007	0.008
	*t*	8.385	7.844	8.342	14.026
	*p*	0.000	0.000	0.000	0.000

**Table 5:** Correlation of age and POVA for male patients.

		Zhoukou *n* = 326	Yuncheng *n* = 442	Sanmenxia *n* = 506	Total *n* = 1274
Correlations	Pearson	0.290	0.170	0.206	0.206
	*p*	0.000	0.000	0.000	0.000

*R*		0.290	0.170	0.206	0.206
*R* ^2^		0.084	0.029	0.042	0.042

ANOVA	*F*	29.687	13.078	22.272	56.452
	*p*	0.000	0.000	0.000	0.000

Coefficients	Constant	−0.384	−0.073	−0.217	−0.189
	*B*	0.009	0.005	0.006	0.006
	*t*	5.449	3.616	4.719	7.513
	*p*	0.000	0.000	0.000	0.000

**Table 6:** Correlation of age and POVA for female patients.

		Zhoukou *n* = 565	Yuncheng *n* = 760	Sanmenxia *n* = 853	Total *n* = 2178
Correlations	Pearson	0.245	0.239	0.241	0.244
	*p*	0.000	0.000	0.000	0.000

*R*		0.245	0.239	0.241	0.244
*R* ^2^		0.060	0.057	0.058	0.059

ANOVA	*F*	35.875	45.907	52.585	137.596
	*p*	0.000	0.000	0.000	0.000

Coefficients	Constant	−0.256	−0.248	−0.238	−0.255
	*B*	0.009	0.008	0.007	0.008
	*t*	5.990	6.775	7.252	11.730
	*p*	0.000	0.000	0.000	0.000
